# Psychiatric Comorbidity and Stress in Medical Students Using Neuroenhancers

**DOI:** 10.3389/fpsyt.2021.771126

**Published:** 2021-12-16

**Authors:** Tarek Jebrini, Kirsi Manz, Gabriele Koller, Daniela Krause, Michael Soyka, Andreas G. Franke

**Affiliations:** ^1^Department of Psychiatry, University Hospital, LMU Munich, Munich, Germany; ^2^Faculty of Medicine, Institute for Medical Information Processing, Biometry and Epidemiology (IBE), LMU Munich, Munich, Germany; ^3^Hochschule der Bundesagentur für Arbeit (HdBA), University of Applied Labour Studies, Mannheim, Germany

**Keywords:** neuroenhancement (NE), cognitive enhancement, misuse, students, medicine

## Abstract

**Background:** Pharmacological neuroenhancement (PN) is a common healthcare problem at least among students. PN seems to be associated with stressful situations. There is a lack of data about personal characteristics, comorbidities, and coping strategies regarding stress and factors of resilience in students and medical staff.

**Methods:** A web-based survey about the non-medical use of PN drugs with a focus on neuroenhancement was developed and distributed among medical students throughout Germany; the questionnaire was open in April and May of 2020. The survey contained questions about the use of well-known PN drugs, frequency, special purposes, reasons for the use, psychiatric disorders, use of psychotropic drugs apart from PN purposes, and factors of resilience using the brief resilience scale.

**Results:** Data of 1,159 students of medicine were analyzed. The most frequently used substances for PN were coffee (78.8% lifetime prevalence rate), energy drinks (45.7%), caffeine tablets (24.3%), methylphenidate (5.2%), illicit amphetamines (2.0%), and cocaine (1.7%). 98.4% suspected that PN drug use could lead to addiction. PN drug use specifically for PN was significantly associated with the use of (a) any psychotropic drug (other than neuroenhancers), (b) any psychiatric disorder, and (c) higher values of feeling pressure to perform in professional/students' life and in private life as well as (d) the subjective feeling of pressure to perform to be burdening and (e) harmful to one's own health. PN drug use in general was significantly associated with being less resilient. The use of illicit PN drugs, over the counter drugs and prescription drugs was associated with being less resilient.

**Conclusion:** This study indicates that PN with legal and illegal drugs is a widespread phenomenon among German medical students. Users seem to be more often burdened by psychiatric disorders, especially addictive disorders, the perception of stress, pressure to perform and low levels of resilience. These aspects should be considered in further investigation of PN drug use.

## Introduction

One of the first and most cited articles in the field of pharmacological neuroenhancement (PN) is titled “Look who is doping” (1). It is based on an online poll that the journal Nature distributed among their readers to find out more about the use of methylphenidate, modafinil, and beta-blockers with the particular intention to enhance one's own cognitive performance. This phenomenon of using so-called “smart drugs” has lots of synonyms e.g., brain doping, academic performance enhancement, cognitive enhancement or pharmacological neuroenhancement and is mostly defined as the non-medical use of divergent psychoactive substances to increase vigilance, attention, concentration or memory by healthy subjects (2–4). The above mentioned Nature article described that 20% of the 1,400 participants had used at least one of the aforementioned drugs with the intention of neuroenhancement (1). Meanwhile there are several national and international publications about the use of prescription as well as illicit drugs for PN showing lifetime prevalence rates of 1 up to 20% depending on the drugs assessed, the survey methods used and other factors among divergent groups of participants (5–10).

In many epidemiological studies, survey participants are not characterized in an adequate way. Only few studies deal with specific target groups. In Germany, a first study about PN was published in 2011 and dealt with pupils and university students of three faculties (medicine, pharmacy, economics) and demonstrated that only 0.8% of the surveyed 500 students had ever used prescription and only 2.9% illicit stimulant drugs for PN purposes (11, 12). For caffeine significantly higher prevalence rates of more than 10% for caffeine tablets and more than 80% for coffee, respectively, were shown (12, 13). However, the above mentioned data of 2011 were raised based on a small sample of medical students in lecture rooms meaning that there was a severe lack of anonymity, possibly influencing the overall result. A similar sample with 206 French medicine and pharmacology students was examined though in an online survey, showing higher prevalence rates of 5.8% for the use of illicit pharmacological neuroenhancers (14). This study with its rather small sample size ensured participants' anonymity. In Italy, a PN study aiming at medical students was conducted in 2018, surveying 363 students with an online questionnaire (15). The authors reported, that only 0.6% of the participants used illicit drugs for PN within a month, while the rates of caffeine use was significant higher. An online poll conducted in Brazil surveyed a small sample of *N* = 152 5th and 6th year medical students for their use of alcohol and methylphenidate (MPH). The findings showed that 23% of the participants used MPH for PN, while the prevalence in the group of 6th year medical students was twice as high as the prevalence in the group of 5th year medical students (16).

Studying medicine is very demanding and associated with high personal distress (17). Numerous studies reported elevated prevalence rates for symptoms of burnout, anxiety, and depression among medical students (18–22). These stress levels can be associated with an elevated risk for health problems, such as substance abuse. Various studies investigated the use of psychotropic substances in the cohort of medical students, demonstrating high prevalence rates of misuse for alcohol, nicotine, and illicit drugs (23–26).

Coping with stressful situations was frequently reported as the underlying motive for using PN drugs (9, 27–31). Furthermore, Bagusat et al. discussed that the individual ability to recover from stress may decrease the risk of using any substance for PN (27). This hypothesis leads to the assumption that PN drug use could be more prevalent in subjects who are less resilient to stress. Yet this rather vague hypothesis has not been investigated so far in students or even medical students with its special situation outlined above.

However, the concept of “resilience” seems to be associated with the stress coping concept. Resilience refers to the phenomenon that many people maintain mental health or only temporally become mentally ill despite significant demands such as stress and/or other adversities (32, 33). Resilience is predominantly understood as the ability to recover from stress or metaphorically as the ability to “bounce back” (34). In order to measure resilience, there are several instruments [e.g., Dispositional Resilience Scale (DRS), Connor-Davidson Resilience Scale (CD-RISC), etc.] (35, 36). Smith et al. developed the Brief Resilience Scale (BRS) (34) that was translated in various languages such as German. It was evaluated as well as validated among a population based and representative sample in/for Germany (*N* = 1,128 German adults) (37).

Summing up, there is a lack of studies regarding PN drug use among specific students or faculties. Furthermore, the aspects of suffering from stress and psychiatric disorders and resilience are scarcely investigated in the context of PN drug use. Therefore, the present web-based study (using the web-based survey tool “soscisurvey”) was designed to learn more about the knowledge and the use of PN drugs as well as the perception of pressure, stress, and psychiatric disorders combined with factors of resilience by surveying medical students throughout Germany.

## Materials and Methods

The present study was designed as an online survey using the tool “soscisurvey,” which is a professional survey tool enabling anonymized surveys (www.soscisurvey.com).

Information about the study was distributed in March and April of 2020 among closed Facebook groups of medical students throughout Germany. Announcements contained a hyperlink to the respective survey on the soscisurvey homepage. The survey was open in April and May of 2020.

### Data Acquisition

Data was acquired using a self-designed survey and a standardized questionnaire (brief resilience scale, BRS). The survey was presented as an online poll to ensure a high degree of privacy and anonymity to all participants.

The questionnaire was entitled as “Scientific survey about mental improvement.” It started with a general introduction about PN, the duration and the scope of the survey. After that declaration about anonymity in general, anonymous data handling and storage as well as the exclusive scientific use of the collected data followed. PN was defined as “the non-medical use of divergent substances to increase mental abilities.”

Subsequently, participants were asked to answer a set of questions about their demographic characteristics (gender, age, country, postal code, university, and study subject) followed by questions about study success and satisfaction, diagnosed psychiatric disorders, reasons and/or prerequisites for PN drug use, and source of knowledge of PN drug use. Subsequently, a set of the following well-known PN drugs was shown: coffee, energy drinks, caffeine tablets, black tea, Ginkgo biloba, methylphenidate, amphetamines, atomoxetin, modafinil, antidementia drugs, ecstasy, ephedrine, cocaine, antidepressants. Participants were asked for frequency of using the above mentioned drugs (never, once per year/month/week, even more frequently). Free space enabled participants to add specific substances.

Next, the six questions of the BRS were used to raise data about aspects of participants' status of resilience (item 1: “I tend to bounce back quickly after hard times,” item 2: “I have a hard time making it through stressful events,” item 3: “It does not take me long to recover from a stressful event,” item 4: “It is hard for me to snap back when something bad happens,” item 5: “I usually come through difficult times with little trouble,” item 6: “I tend to take a long time to get over set-backs in my life”) (34, 38): Resilience can to understood as an ability to recover (from stress) or the ability to “bounce back” (34). However, this definition was not given to the participants. The BRS uses 5 step Likert scales for each of the six statements (completely agree, agree, neutral, disagree, completely disagree). The resilience was calculated as the mean of the six items Higher BRS scores correspond to higher resilience and vice versa.

**Figure 1:**
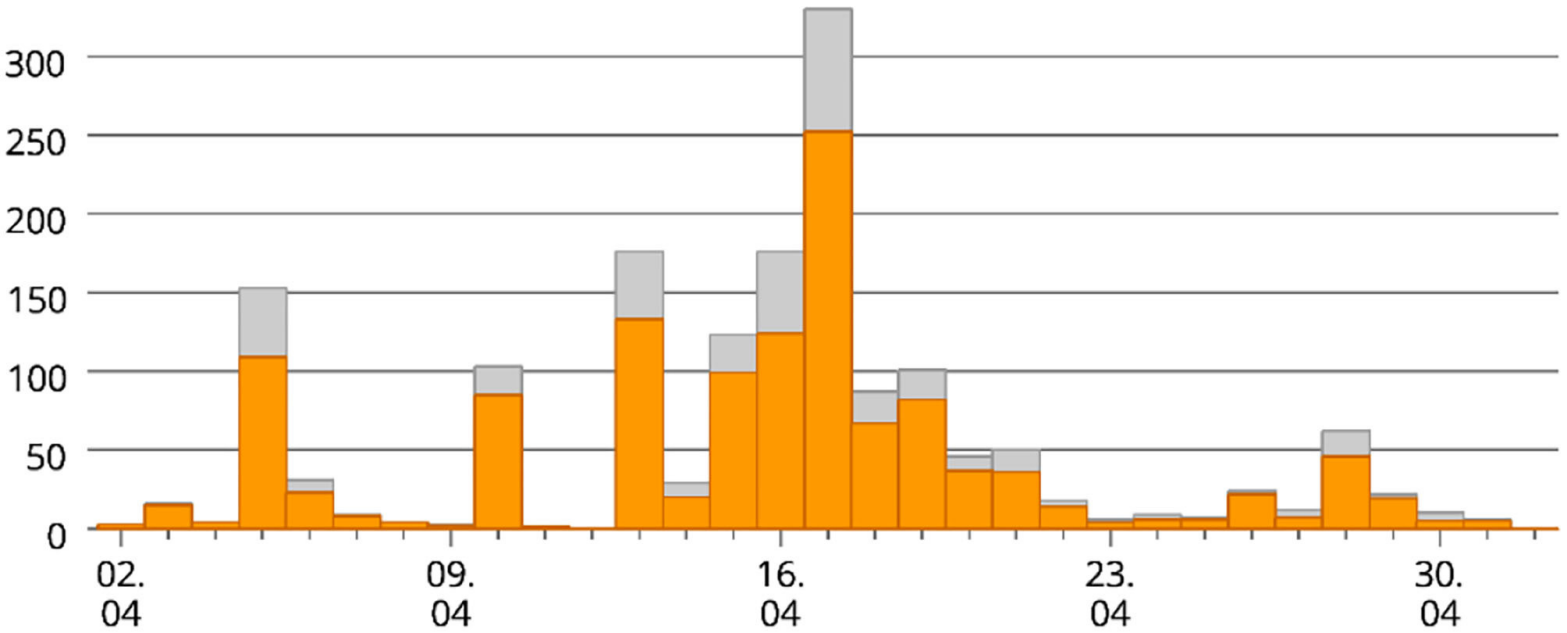
Process of participation. Horizontal axis, data acquisition period between April and May 2020; vertical axis, number of participants; orange, complete data; gray, incomplete data.

The process of participation is shown in Figure 1, where the number of answered surveys over time is shown.

### Data Analysis

Data were collected and stored in the “soscisurvey” database on servers in Munich. Statistical analyses were performed with SPSS for Windows, version 22.0 and R version 3.6.3.

Continuous variables were summarized by their mean M and standard deviation SD. Logistic regression was applied to search for associations between different independent factors and the dependent factors of PN drug use/illegal drug use/OTC drug use/prescription drug use/drug use for non-PM purposes, respectively. Odds ratios OR are reported together with their 95% confidence intervals.

The PN drugs were grouped as follows: over the counter (OTC) PN drugs = coffee, energy drinks, caffeine tablets, cola drinks, black/green tea, Ginkgo biloba; prescription PN drugs: methylphenidate, amphetamine preparations, Atomoxetine, Modafinil, antidementives, antidepressants; illicit PN drugs: Ecstasy (MDMA), ephedrine, cocaine, illegal amphetamines (e.g., Speed). PN drug use in general was defined as the reported use of any of the PN drugs. Use of OTC PN drugs, PN prescription drugs and PN illicit drugs was analogously defined as the reported use of any of the drugs in the above defined groups. Non-PM drug use was defined as use of any of the following drugs for non-PM purposes: alcohol, nicotine, cannabis, illegal amphetamines, cocaine, benzodiazepines, hallucinogens, opioids.

The results are reported using unadjusted *p*-values without considering adjustments for multiple comparisons. The significance level was set at 0.05.

### Ethics Statement

The study was performed according to the Declaration of Helsinki. Participants gave their informed consent by clicking on a button after reading a short introductory paragraph and by pressing the button “done” at the end of the survey. This procedure as well as the whole study was approved by the responsible ethics committee of the Ludwig Maximilians University (number 18–550 UE).

## Results

### Participants' Characteristics

In total, 2,632 students took part in the survey by visiting the survey's URL and 1,247 participants completed the questionnaires. Eighty-eight participants were excluded because of studying abroad or being no medical student (students of psychology and dentistry). Therefore, data of *N* = 1,159 participants are the basis of all further calculations.

Most of the participants were females (72.3%, *N* = 838). Among the 1,159 participants, 17.9% (*N* = 207) reported having been diagnosed with a psychiatric diagnosis, 5.4% (*N* = 63) stated being currently treated with a prescription drug for a mental illness. Nearly half of the participants had already reached the clinical part of the study course (48.6%, *N* = 563). The majority of participants (83.4%, *N* = 967) reported being satisfied with their university success to date. Most participants came from the region around Munich (13.3%) followed by regions around Würzburg (5.5%), Freiburg (5.3%), Ulm (4.6%), and Marburg (3.9%). For further characteristics see Table 1.

**Table 1:** Overview of participants' characteristics.

**Participants' characteristics**	
Sex	Male: 27.5% (*N* = 319) Female: 72.3% (*N* = 838) Not specified: 0.2% (*N* = 2)
Highest academic degree	High school leaving certificate: 30% (*N* = 348) First state examination: 48.6% (*N* = 563) Second state examination: 12.2% (*N* = 141) Third state examination: 4.7% (*N* = 54)
Postal code cluster	Munich (80xxx−81xxx): 13.3% (*N* = 154) Würzburg (97xxx): 5.5% (*N* = 64) Freiburg (79xxx): 5.3% (*N* = 61) Ulm (89xxx): 4.6% (*N* = 53) Marburg (35xxx): 3.9% (*N* = 45)
Hours of work per week for studying medicine	Mean 16 h, SD ±1.4 h
Being satisfied with professional success (at university)	Yes: 83.4% (*N* = 967) No: 16.6% (*N* = 192)
Being one of the most successful students (better than 80% of all students)	5.1% (*N* = 59)

### Substance Use for Pharmacological Neuroenhancement

Of all participants, 46.9% (*N* = 543) stated that they would use OTC drugs to specifically enhance their cognitive performance, while 4.8% (*N* = 56) stated that they would consider using illegal substances for performance enhancement. 98.4% (*N* = 1,141) of the participants agreed with the statement that the use of prescription and/or illegal drugs, which are consumed specifically to enhance performance, can lead to addiction.

Regarding the question of having used PN drugs, 90.1% (*N* = 1,044) of the participants stated to have ever used PN drugs. Prevalence rates differed regarding the substances used: Coffee was the most frequently used substance (drink) for PN purposes; 78.8% (*N* = 913) stated to have used coffee for PN purposes; and 49.3% (*N* = 571) have reported multiple consumption during the last week. In contrast, atomoxetine was the least frequently used PN drug being used once by only one participant.

In general, legal OTC drugs were most frequently used for PN purposes (89.2%, *N* = 1,041), followed by the use of prescription drugs (8.8%, *N* = 102) and illegal drugs (3.0%, *N* = 35). However, prescription as well as illicit drug use was infrequent. Although, caffeine tablets that are only sold in pharmacies in Germany without the necessity of a physicians' prescription have been used by 24.3% (*N* = 282) and energy drinks by 45.7% (*N* = 530). MPH is the most frequently used prescription drug (5.2%, *N* = 60) and amphetamines (2.0%, *N* = 23) and cocaine (1.7%, *N* = 20) the two most frequently used illicit drugs. More data are presented in Table 2.

**Table 2:** Use of PN drugs and frequency of this use.

**Use of any surveyed substance**	**Never used**	**Ever used**			
		**Within last 12 months**	**Within last 30 days**	**Once within last 7 days**	**Multiple within the last 7 days**
Coffee	*N* = 246 (21.2%)	*N* = 91 (7.9%)	*N* = 136 (11.7%)	*N* = 115 (9.9%)	*N* = 571 (49.3%)
Energy drinks	*N* = 629 (54.3%)	*N* = 255 (22.0%)	*N* = 128 (11%)	*N* = 40 (3.5%)	*N* = 107 (9.2%)
Caffeine tablets	*N* = 877 (75.7%)	*N* = 139 (12.0%)	*N* = 61 (5.3%)	*N* = 32 (2.8%)	*N* = 50 (4.3%)
Cola	*N* = 477 (41.2%)	*N* = 173 (14.9%)	*N* = 253 (21.8%)	*N* = 128 (11.0%)	*N* = 128 (11.0%)
Black/green tea	*N* = 426 (36.8%)	*N* = 115 (9.9%)	*N* = 199 (17.2%)	*N* = 172 (14.8%)	*N* = 247 (21.3%)
Ginkgo biloba	*N* = 1,077 (92.9%)	*N* = 38 (3.3%)	*N* = 20 (1.7%)	*N* = 7 (0.6%)	*N* = 17 (1.5%)
Methylphenidate	*N* = 1,099 (94.8%)	*N* = 33 (2.8%)	*N* = 8 (0.7%)	*N* = 3 (0.3%)	*N* = 16 (1.4%)
Amphetamine preparations	*N* = 1,150 (99.2%)	*N* = 6 (0.5%)	*N* = 2 (0.2%)	*N* = 1 (0.1%)	–
Atomoxetine	*N* = 1,158 (99.9%)	*N* = 1 (0.1%)	–	–	–
Modafinil	*N* = 1,138 (98.2%)	*N* = 11 (0.9%)	*N* = 2 (0.2%)	*N* = 3 (0.3%)	*N* = 5 (0.4%)
Antidementia drugs	*N* = 1,156 (99.7%)	*N* = 2 (0.2%)	*N* = 1 (0.1%)	–	–
Ecstasy (MDMA)	*N* = 1,147 (99%)	*N* = 8 (0.7%)	*N* = 4 (0.3%)	–	–
Ephedrine	*N* = 1,147 (99%)	*N* = 9 (0.8%)	*N* = 3 (0.3%)		
Cocaine	*N* = 1,139 (98.3%)	*N* = 16 (1.4%)	*N* = 4 (0.3%)	–	–
Illegal amphetamines (e.g., Speed)	*N* = 1,136 (98.0%)	*N* = 15 (1.3%)	*N* = 8 (0.7%)	–	–
Antidepressants	*N* = 1,131 (97.6%)	*N* = 6 (0.5)	*N* = 4 (0.3%)	*N* = 1 (0.1%)	*N* = 17 (1.5%)

For the PN drug use *in general* and the feeling of being satisfied with one's own professional success at university the odds ratio was OR = 0.45 (95% confidence interval: 0.22, 0.84), *p* = 0.0195) pointing toward significantly decreased odds for PN use for satisfied participants. The association between general PN drug use and being among the most successful students did not gain statistical significance [OR = 0.66 (0.42, 1.07), *p* = 0.0824]. However, also here the odds for PN drug use are decreased in students rating themselves among the best 20%. There were no gender differences in PN drug use in general [OR = 0.86, (0.57, 1.32), *p* = 0.4690 for males compared to females].Regarding the association of using *illicit PN drugs* and being satisfied with the own success at university [OR = 0.78 (0.36, 1.98), *p* = 0.5800] as well as between the use of illicit PN drugs and being among the most successful students [OR = 1.24 (0.49, 2.74), *p* = 0.6110] there were no statistically significant results. However, compared to female students male students had higher odds for use of illicit PN drugs [OR = 2.02 (1.00, 3.98), *p* = 0.0437].

Regarding the aspect of being satisfied with one's own professionally success (at university) there was a significant association with the use of *prescription PN drugs* [OR = 0.43 (0.28, 0.69), *p* = 0.0004] showing decreased odds for prescription drug use in satisfied students. For the aspect of using prescription PN drugs and being among the most successful students there was no statistically significant association found [OR = 0.99 (0.56, 1.66), *p* = 0.9640]. A gender effect for prescription PN drug use was found with males having higher odds for the use [OR = 2.57 (1.70, 3.88), *p* < 0.0001]. For the use of *OTC drugs* being satisfied with professional success at university showed a statistically significant association [OR = 0.49 (0.24, 0.89), *p* = 0.0285]. Being among the most successful students was not associated with the use of OTC drugs [OR = 0.69 (0.44, 1.11), *p* = 0.1120], neither was participant's gender [OR = 0.85 (0.57, 1.30), *p* = 0.4520 for males compared to females].

### Substance Use NOT for Pharmacological Neuroenhancement

Beyond the set of questions of using substances for the purpose of PN, a further set of questions asked for the use of divergent psychotropic drugs explicitly *not* for PN but recreational use. Answers of the 1,159 participants show that the most frequently used psychotropic drug not used for PN was alcohol with a lifetime prevalence of 90.3% (*N* = 1,047) followed by nicotine (31.7%, *N* = 367). The prevalence rate of the use of cannabis (34.5%, *N* = 400) was higher compared to the lifetime prevalence of nicotine. The use (ever/lifetime) of illicit amphetamines was stated by 9.7% of the participants (*N* = 112), respectively, followed by cocaine (6.6%, *N* = 77). The most infrequently used illicit drugs were opioids (0.7%, *N* = 8). For further data see Table 3.

**Table 3:** Use of psychoactive substances explicitly NOT for PN.

**Use of any surveyed substance**	**Never used**	**Ever used**			
		**Within last 12 months**	**Within last 30 days**	**Once within last 7 days**	**Mutiple within the last 7 days**
Alcohol	*N* = 112 (9.7%)	*N* = 96 (8.3%)	*N* = 364 (31.4%)	*N* = 387 (33.4%)	*N* = 200 (17.3%)
Nicotine	*N* = 792 (68.3%)	*N* = 119 (10.3%)	*N* = 100 (8.6%)	*N* = 37 (3.2%)	*N* = 111 (9.6%)
Cannabis	*N* = 759 (65.5%)	*N* = 254 (21.9%)	*N* = 91 (7.9%)	*N* = 30 (2.6%)	*N* = 25 (2.2%)
Illegal amphetamines	*N* = 1,047 (90.3%)	*N* = 80 (6.9%)	*N* = 30 (2.6%)	*N* = 2 (0.2%)	–
Cocaine	*N* = 1,082 (93.4%)	*N* = 59 (5.1%)	*N* = 17 (1.5%)		*N* = 1 (0.1%)
Benzodiazepines	*N* = 1,105 (95.3%)	*N* = 36 (3.1%)	*N* = 16 (1.4%)	*N* = 1 (0.1%)	*N* = 1 (0.1%)
Hallucinogens	*N* = 1,118 (96.5%)	*N* = 36 (3.1%)	*N* = 5 (0.4%)	–	–
Opioids	*N* = 1,151 (99.3%)	*N* = 6 (0.5%)	*N* = 1 (0.1%)	*N* = 1 (0.1%)	–

Regarding the association of using substances for PN and using psychoactive substances NOT for PN, the majority of the participants used substances for both purposes (*N* = 977, 84.3%). Only *N* = 32 participants (2.8%) did not use drugs for PN or other purposes (*N* = 32). 7.2% (*N* = 83) of all participants used drugs for non-PM purposes only and 67 participants (5.8%) stated to have used drugs for PN purposes only. There were statistically significantly higher odds for PN use in the participants using drugs for non-PN purposes compared to participants without non-PN drug use [OR = 5.41 (3.34, 8.65), *p* < 0.0001]. Use of psychoactive substances explicitly not for PN was not associated with satisfaction with professional success at the university [OR = 1.21 (0.70, 2.02), *p* = 0.4630] or with being among the best students [OR = 0.97 (0.58, 1.73), *p* = 0.9230]. No gender differences in non-PN use was found [OR = 1.21 (0.76, 1.99), *p* = 0.439 for males compared to females].

### Mental Disorders

The majority of participants had no psychiatric diagnoses (82.1%, *N* = 952). However, 11.7% (*N* = 136) of the participants were diagnosed with depression in the past, 1.4% (*N* = 16) with burnout syndrome and 2.0% (*N* = 23) with ADHD. “Other disorders” were named by 7.3% (*N* = 85) of the participants, however, no further specification was given (Table 4).

**Table 4:** Diagnoses of participants' mental disorders.

**Diagnosis**	**Affected participants**
No psychiatric diagnosis	*N* = 952 (82.1%)
Depression	*N* = 136 (11.7%)
Burnout	*N* = 16 (1.4%)
ADHD/ADS	*N* = 23 (2.0%)
Others	*N* = 85 (7.3%)

*ADHD, attention deficit hyperactivity disorder*.

The group of diagnosed participants showed a significantly higher rate of PN drug use than non-diagnosed participants [OR = 1.97 (1.11, 3.84), *p* = 0.0313]. However, the use of psychoactive drugs not for PN reasons was not statistically significantly associated with presence psychiatric diagnoses [OR = 1.14 (0.67, 2.06), *p* = 0.645].

### Pressure to Perform

Using six step Likert scales (between 0 = nothing/“not a bit” and 5 = very strong) participants were asked to what extent they felt pressure to perform regarding studying at university. The question leads to a mean of *M* = 4.45 (SD: 1.216). Regarding the aspects of feeling pressure to perform in private life, this question leads to a mean of M = 3.27 (SD: 1.339). Considering pressure to be burdening shows a mean of M = 3.910 (SD: 1.365) and considering pressure to be harmful to health led to a mean of M = 4.22 (SD: 1.343). The question of feeling a certain pressure to perform has risen during the last years resulted in a mean of M = 4.20 (SD: 1.492).

Regarding the use of psychoactive drugs for PN and factors of pressure to perform, there were statistically significant findings for feeling pressure to perform in professional life (studying at university) [OR = 1.27 (1.09, 1.48), *p* = 0.0022 per increase of one step in the Likert scale] and in private life [OR = 1.17 (1.01, 1.38), *p* = 0.0366 per one step increase in the Likert scale], for feeling rising levels of pressure to perform [OR = 1.20 (1.06, 1.36), *p* = 0.0042 per one step increase in the Likert scale], considering pressure to perform to be burdening [OR = 1.26 (1.10, 1.45), *p* = 0.0013 per one step increase in the Likert scale] and considering pressure to perform to be harmful to one's own health [OR = 1.21 (1.05, 1.39), *p* = 0.0069 per one step increase in the Likert scale].

### Coping Behavior and Resilience

To assess resilience, all participants filled out the Brief Resilience Scale (mean sum score *M* = 20.72, SD = 4.28). Furthermore, participants stated different strategies to cope with stressful situations and the feeling or even fact of pressure to perform.

Most frequent individual strategies to cope with stress were watching films and TV series (*N* = 948; 81.8%), commitments in social projects (*N* = 327; 28.2%) as well as having sex (*N* = 586; 50.6%), meetings with family (*N* = 765; 66%), and partying (*N* = 687; 59.3%).

Regarding resilience, lower scores of the BRS scale mean less resilience against stress. For the PN drug use in general the there was a statistically significant association with the BRS score [OR = 0.89 (0.85, 0.94), *p* < 0.001]. Thus, per one step increase in the BRS score the odds for PN drug use decreased by 11%.Regarding the use of illicit drugs for PN, no statistically significant association with BRS scores was found [OR = 1.01 (0.94, 1.10), *p* = 0.7860]. However, a significant association was found between the use of OTC drugs for PN and BRS values [OR = 0.90 (0.85, 0.94), *p* < 0.0001] pointing toward lower odds for OTC drug use for higher resilience score. A similar association was found for the use of prescription drugs for PN and resilience scores [OR = 0.92 (0.88, 0.97), *p* = 0.0005]. Regarding the association of BRS score and using psychoactive substance NOT for PN, there was no significant association [OR = 1.01 (0.97, 1.06), *p* = 0.5670].

## Discussion

The phenomenon of neuroenhancement is a highly relevant issue in the context of medical students especially regarding legal OTC drugs. However, illicit and prescription drugs are used for PN purposes in Germany, too. Furthermore, the pattern of using PN drugs in general is associated with the use of psychoactive drugs not for PN purposes. Beyond that, medical students using PN drugs were more likely to feel stress and regarded stress to be harmful and burdening. So far resilience has not yet been explored in this population group in regard to PN. In a study investigating university students from UK and Ireland, resilience was interpreted as a low lifetime use of PN in a context were there was a high awareness of and interest in PN (39). This study indicates that low levels of resilience are associated with the risk of using PN drugs. Assuming that low levels of resilience are associated with higher prevalence rates of PN drug use, individuals with this pattern could be (or at least feel to be) more exposed and even more vulnerable to negative effects of substance abuse and performance pressure.

Prevalence rates of PN drug use among divergent populations show a large range between lower than 1% lifetime prevalence rate especially for prescription and/or illicit drugs (11) and more than 20% 1-year prevalence rate for “taking drugs only to improve their cognitive performance and not to treat underlying mental disorders” (8).

In our present student sample, the results can be described as being “in-between” compared to other studies. While some studies reported higher rates for the use of prescription and illicit drugs for PN (14, 16), other studies report lower rates than the results demonstrated in the present study (11, 15). The different results may be due to the type of drug(s) assessed (OTC drugs, prescription drugs, illegal drugs), the assessed participants (students, physicians, other homogenous/heterogenous groups) and especially the degree of anonymity of the study design (paper and pencil survey, web based survey, anonymizing techniques) as well as the year of investigation. For example, Franke et al. assessed the use of caffeinated beverages among students using a web based survey design and received high double-digit percentages (13). However, the same group around Franke received a lifetime prevalence lower than 1% among students assessing the use of prescription stimulants in a paper and pencil survey among students sitting next to each other (11). Dietz et al. used an anonymizing survey technique (randomized response technique, RRT) among a student sample and received a 1-year prevalence rate of more than 20%, respectively.

The present study revealed a significant proportion of participants using PN drugs with the aim to increase cognitive performance. Furthermore, there was a significant number of participants using psychotropic substances not for PN. In this context, a significant association between those who used PN drugs for PN and those who used divergent psychotropic substances not for PN could be demonstrated. This is in line with previous findings of a very small study about characteristics of students using stimulants such as amphetamines and methylphenidate for PN (40). This study revealed that the group of stimulants' using students for PN used illicit substances (not for PN) more frequently with significantly higher rates of diagnoses of substance misuse of alcohol and cannabis (40). The diagnosis of a substance use disorder was raised using a structured clinical interview part 1 (SKID-I). Beyond that, the significant association between PN drug use and any diagnosis of a substance use disorder is enlarged in the present study by the significant association between using PN drugs for PN purposes and being diagnosed with any psychiatric disorder. This association seems quite clear as neuropsychiatric disorders clinically manifest often with cognitive disturbances such as impairments of higher executive functions, regulation of attention, aberrant learning (41). At least to the authors' knowledge, there are no studies directly measuring the use of PN in the population with a mental disorder.

For the source of knowledge and acquisition of PN a recent meta-analysis showed that PN drugs are largely obtained from friends, family and *via* the Internet. Based on these aspects, it has been suggested, that PN is mainly occurring among “healthy individuals, mainly students without any diagnosed cognitive disorders” (42). It can be questioned if the acquisition of PN drugs by friends, families and the internet reveals that PN drug users are “healthy” and without any (psychiatric) diagnosis.

The present study does not only show the use of PN drugs to obtain a better cognitive performance but also depicts that the vast majority of the participants feel a pressure to perform. The highest pressure was felt regarding professional life followed by private life. These results are in line with an enormous pressure to perform among physicians (29). This could mean that a certain pressure to perform starts during studying medicine and continues in professional life.

Furthermore, the above mentioned study among surgeons revealed that the use of illegal and prescription PN drugs to increase performance was based on pressure to perform (29). This result is underlined by the present findings.

Furthermore, the fact or even the feeling of pressure to perform being high among the study participants is accompanied by divergent coping strategies. Beside the coping strategies watching films/TV, commitments in social projects, etc. another destructive coping strategy is the use of psychotropic substances not specifically for PN. Our study revealed a relatively high proportion of participants using divergent psychotropic drugs, especially alcohol, nicotine, and cannabis. This is in line to previous studies that have demonstrated drug use as a coping strategy for stress (43–45). Perhaps there is not just the one and only neuroenhancement drug. It seems more likely that there is a whole heterogenous set of interventions including behavioral strategies to improve performance (46). Beyond that, the concept of resilience is getting important in this context. The concept of resilience is defined as the outcome of a process of successfully dealing with or adapting to stressors (47, 48). Therefore, the present study measures participants' “degree” to recover from stress or to “bounce back.” Unfortunately, there is only one study which has tried to combine the concept of resilience to the phenomenon of PN drug use (27): Bagusat et al. suggest that the non-medical use of prescription drugs for PN drugs appears to be more prevalent in subjects who are less resilient to stress. This is systematically investigated and underlined by the present study results showing (a) PN drug use in general for PN, (b) illicit drug use for PN, (c) the use of OTC drug for PN as well as the use of (d) prescription drugs for PN are significantly associated with the participants' characteristic of being less resilient.

In the present study we used the German version of the Brief Resilience Scale (BRS) to assess resilience among the study participants. The original of the BRS was introduced by Smith et al. in 2008 (34). Even though there are a few instruments “asserting” to measure resilience (38, 49), in systematic comparisons with other resilience scales, the BRS received the highest ratings concerning internal consistency, convergent and discriminant validity (49). For the German-speaking participants, we used the German version of the BRS. This version was validated by Bagusat et al. in 2018 (27). Therefore, we evaluated the used German BRS to be the best mean to measure resilience.

### Limitations

The present study also has some limitations that are worth to be mentioned. The study design has to be considered to be the main limiting aspect: The aim was to assess PN drug use among medical students and associations with substance use and resilience in Germany using an online design. Even if the announcements were explicitly placed on digital platforms where only medical students should be expected, it cannot be assured that only German medical students took part in the survey. In online studies, neither the response rate nor the type of participants or a double participation can be controlled. Furthermore, the study was done by the Ludwig Maximilians University of Munich (LMU) in the south of Germany, so that it could be assumed that a higher proportion of students of the LMU may have participated in this study. This bias cannot be controlled by online surveys, too.

Another important aspect deriving from the above mentioned aspect is the limitation of generalizing the results, because the study sample cannot be considered as representative for the whole group of German medical students.

However, this study raised confirmatory results about the prevalence rate of PN drug use and raised preliminary data about the association of PN drug use with the use of divergent psychoactive substances not for PN, the feeling or even fact of being “stressed” by the pressure to perform and the fact of resilience.

## Conclusion

The drive to improve cognitive performance can be seen as a common phenomenon in students. The present study demonstrates that PN with legal and illegal drugs is quite common in German medical students. Factors such as psychiatric disorders (especially addiction), the feeling of stress, pressure to perform, and less resilience are associated with the use of PN drugs. Helping students to adequately reduce these factors might diminish the overall use of PN drugs.

## Data Availability Statement

The raw data supporting the conclusions of this article will be made available by the authors, without undue reservation.

## Ethics Statement

The studies involving human participants were reviewed and approved by Ethics Committee of the Ludwig Maximilians University 18-550 UE. The patients/participants provided their written informed consent to participate in this study.

## Author Contributions

TJ, GK, and AF made substantial contributions to the conception and design of the study, the data acquisition, analysis and interpretation of data, and the preparation of the manuscript. TJ was the leading part of data acquisition, data analysis, and was supported by KM. MS, GK, DK, and AF made substantial contributions to the interpretation of data and the preparation of the manuscript. All authors proofread and accepted the final version of the manuscript.

## Conflict of Interest

The authors declare that the research was conducted in the absence of any commercial or financial relationships that could be construed as a potential conflict of interest.

## Publisher's Note

All claims expressed in this article are solely those of the authors and do not necessarily represent those of their affiliated organizations, or those of the publisher, the editors and the reviewers. Any product that may be evaluated in this article, or claim that may be made by its manufacturer, is not guaranteed or endorsed by the publisher.
